# Soil Nutrients Rather than Heavy Metals Dominate the Succession of Microbial Communities in Subtropical Coal Gangue Piles: A Case Study from a Successional Gradient in Southern China

**DOI:** 10.3390/microorganisms14102322

**Published:** 2026-10-12

**Authors:** Hanchang Zhou, Tian Wang, Ziying Zhang, Yi Li, Quanquan Jia, Lin Yu, Xiaobao Kuang

**Affiliations:** Carbon Sink Centre, Jiangxi Academy of Forestry, Nanchang 330013, China; zhouhanchang2022@163.com (H.Z.); wangxiuzhi3712@163.com (T.W.); zhangziying_lky@163.com (Z.Z.); liyi6405379@163.com (Y.L.); auu2132057@163.com (Q.J.); yulin0417@163.com (L.Y.)

**Keywords:** subtropical region, coal mine gangue, microbial interaction network, nitrogen and phosphorus, heavy metal fractions

## Abstract

The natural revegetation of coal gangue piles relies on microbial community succession, yet limiting factors in subtropical regions remain unclear, hindering the acceleration of natural revegetation. This study examined coal gangue tailings of varying successional ages in southern China to identify the factors that limits on microbial succession. Soil properties, microbial diversity, community structure, network complexity, and heavy metal fractions were analyzed across successional stages. Results show a progressive soil acidification anda shift from carbon/nitrogen limitation to phosphorus limitation as succession progresses. Bacterial richness peaked at middle stage and then declined, while fungal richness shows no significant difference among different stages. Bacterial community structure changed sharply in early successional stage while fungal structure in the mid-stages. The complexity of microbial cooccurance network initially decreased then increased. Heavy metals mostly accumulated in stable residual fractions during succession, which has only limited toxic effects. The nutrient limitation that shift from C/N to P was the dominant factor shaping core functional modules and community composition, though dissolved organic carbon and few heavy metal fractions were also the main driving factors of microbial network complexity. These findings indicate that microbial succession is constrained more by nutrient availability than by heavy metal toxicity, with early stages limited by C/N sources and later stages limited by phosphorus. This provides a basis for targeted management to accelerate natural recovery of coal gangue piles.

## 1. Introduction

With the rapid development of global industrialization, the demand for coal has sharply increased, generating substantial amounts of waste such as coal gangue during mining and processing [1]. Most of this waste is simply piled up in stockpiles. In China alone, coal mining produces approximately 750 million tons of coal gangue annually, resulting in nearly 6 billion tons of accumulated waste piles [1,2]. These coal gangue piles not only occupy vast areas of potential industrial, agricultural, and natural forest land but also release pollutants such as heavy metals into the surrounding environment, threatening ecosystem security and the health of local residents [2,3]. Managing coal gangue tailings piles and restoring natural vegetation are important parts of environmental protection [3,4].

In subtropical regions, due to relatively hot and wet conditions, the weathering of coal gangue and the release of pollutants occur more rapidly, making restoration efforts even more urgent [2]. Methods for rehabilitating coal gangue piles include soil replacement and natural recovery [5]. The former involves high engineering costs, whereas the latter is more economically feasible and often leads to better long-term restoration outcomes [6,7]. Moreover, many coal mines in subtropical regions are located in developing and less developed countries, where natural restoration aligns best with their economic conditions and development needs, particularly under the current global economic downturn [5]. However, natural restoration typically requires a specific process of community development and is relatively slow in terms of landscape formation. For instance, in southern China, it takes over 20 years for coal gangue piles to naturally recover into visually dense woodland, and more than 50 years to develop into mature forests [8]. Therefore, accelerating the succession of tailings communities is a key challenge in enhancing the efficiency of natural restoration [5,8].

Community succession typically progresses from bare ground to climax vegetation communities [8]. However, a growing body of evidence suggests that soil microbial succession precedes vegetation succession [9,10,11]. By establishing rhizosphere microbial reservoirs and driving key soil nutrient cycles, microbial communities create essential nutrient and soil conditions for plant colonization and subsequent development [9,10]. Vegetation communities can only advance to later successional stages once the microbial community has reached a specific developmental phase [9,10,11]. In tailings areas, the properties of soil microbial communities differ significantly from those of counterparts in adjacent environments. This distinct microbial composition is critical for vegetation colonization in tailings, as it enables adaptation to the shifting environmental conditions across successional stages and supports specific ecological functions [9,10,11]. For instance, fungal communities in heavy metal-laden tailings are particularly adept at secreting polysaccharide-based gels that chelate metal ions, mitigate heavy metal toxicity, and aid host plants in detoxification [10]. Thus, the successional rate of microbial communities directly determines the natural recovery rate of polluted ecosystems.

Microbial communities are complex systems formed by a multitude of microbial taxa interacting in specific proportions, which can be abstractly represented as network structures [12]. Past research has primarily focused on the richness and composition of microbial communities. However, a growing body of evidence indicates that network structure, a fundamental property of microbial communities, is closely linked to the geometric characteristics and assembly patterns of microbial interactions [13,14]. It serves as a key determinant in maintaining and characterizing the multifunctionality and stability of microbial communities, with an influence that often surpasses traditional metrics such as richness and compositional structure [13,14]. Moreover, network structures exhibit distinct characteristics across different successional stages [15]. For instance, these structures are always composed of specialized functional modules—groups of microbial taxa that interact more closely to perform specific ecological functions. Core functional modules frequently assemble around the most critical limiting ecological factors. As key limiting nutrients vary significantly across successional stages, this may lead to differences in the assembly patterns of microbial network structures [12]. Additionally, the complexity of interaction networks can change in response to environmental shifts, such as nutrient accumulation [16]. Therefore, elucidating the successional patterns of microbial interaction networks in tailings ecosystems and identifying the key factors that constrain network evolution at different stages are essential for developing effective strategies to accelerate natural restoration. Nevertheless, studies on the succession of soil microbial network relationships in coal gangue tailings remain scarce, particularly in subtropical regions.

Hence, this study selected sampling sites representing different successional stages in a typical subtropical coal gangue tailings area for in-depth investigation. We hypothesize that tailings generally face conditions such as low nutrient content and heavy metal contamination, both of which likely play major roles in shaping microbial community properties and driving microbial succession. During the early successional stages, heavy metal toxicity is presumed to be the primary limiting factor. At this phase, the bioavailable forms of heavy metals are expected to significantly influence the shaping of community interaction networks, with network structures forming around these stressors. In the mid-to-late successional stages, heavy metal toxicity is anticipated to diminish. Consequently, interaction networks are likely to become increasingly structured around the availability of carbon, nitrogen, and phosphorus sources. In line with soil development patterns in subtropical regions, phosphorus limitation is expected to become progressively more pronounced in the later stages of succession.

## 2. Materials and Methods

### 2.1. Site Selection, Sampling, and Preprocessing

**Figure 1:**
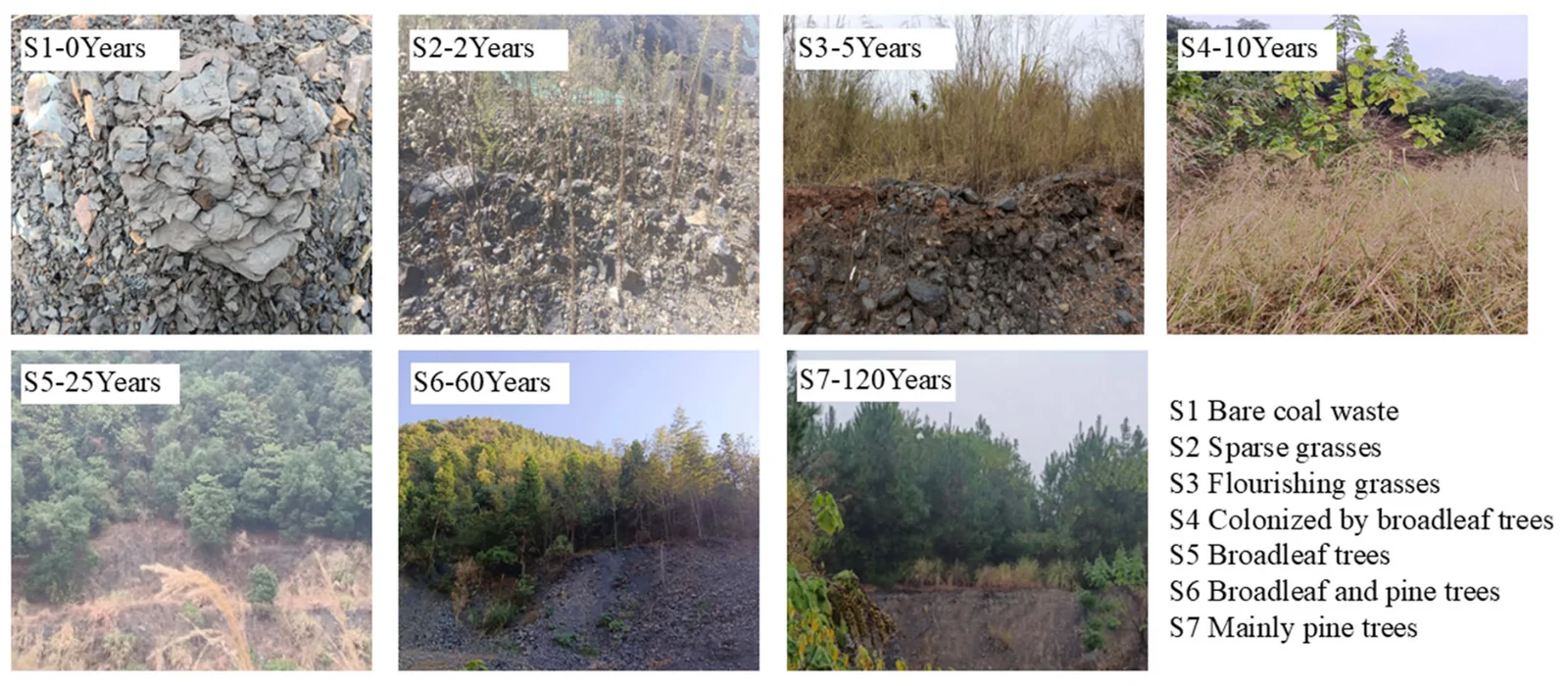
Current vegetation status of the successional study sites. S1 to S7 indicate bare coal gangue (0 years), bare land with few grasses (2 years), grassland (5 years), grassland colonized by broadleaf trees (10 years), broadleaf forest (25 years), mixed broadleaf and coniferous forest (60 years), and coniferous forest (120 years), respectively.

This study focuses on a typical coal gangue tailings area that located within the Anyuan-Wangjiayuan Coal Mine (N 27°36′22.34″, E 113°53′45.96″) in Pingxiang City, Jiangxi Province, China. This mine has been in operation since the late Qing Dynasty, resulting in the accumulation of abandoned coal gangue into artificial mounds. Depending on the time since abandonment, the coal gangue piles at 0, 2, 5, 10, 25, 60, and 120 years exhibit distinct vegetation stages (Figure 1): bare land (S1), sparse grassland dominated by Asteraceae herbs (S2), dense grassland dominated by *Poaceae* grasses (S3), grassland with initial colonization of fast-growing broadleaf trees such as *Paulownia* (S4), broadleaf-dominated woodland (S5), mixed coniferous–broadleaf forest (S6), and conifer-dominated forest (S7). Sites S1 and S2 have almost no plant litter and contain numerous large rocks. Sites S3 and S4 show some herbaceous litter, along with deposits from decomposed moss and fine sediments transported by water or wind. Sites S5 and S6 have a thin layer of woody litter, with surface soil becoming less sandy and loamier. Site S7 features approximately 1 cm of pine needle litter, with further increased loamy texture compared to the previous stage. No artificial forestry or any other vegetation engineering was applied to any of the gangue piles investigated.

Sampling was conducted from 3–6 November 2022. At each site, a 30 m transect (for bare land and grassland) or a 10 m × 10 m quadrat (for woodland) was established in a representative vegetation zone. Within each quadrat, ten 1 m × 1 m sub-quadrats were evenly distributed. Soil samples from the 0–30 cm layer were collected using a soil auger at ten points, mixed, homogenized in situ through a 2 mm sieve, and cleared of stones and plant/animal debris. Approximately 500 g of soil was preserved for physicochemical analysis, and 10 g was stored at −80 °C for molecular biological analysis. Additionally, 100 g of each soil sample was air-dried, ground, and passed through a 250-mesh sieve for heavy metal fractionation analysis. A total of 70 samples were collected from the 7 sites, with each site providing 10 samples.

### 2.2. Basic Nutrient Measurements

Soil total carbon (TC) and total nitrogen (TN) were determined using a Jena C/N analyzer. Dissolved organic carbon (DOC) was extracted with a 1:5 soil-to-water ratio and measured with the same analyzer. Ammonium nitrogen (NH_4_^+^-N) was quantified by the indophenol blue method, and nitrate nitrogen (NO_3_^−^-N) by the dual-wavelength method. Total phosphorus (TP) was measured after digestion with perchloric-sulfuric acid using the spectrophotometry of ammonium molybdate and ascorbic acid method. Available phosphorus (AP) was extracted with a dilute hydrochloric–sulfuric acid mixture and determined by the same method. Soil pH was measured in a 1:2.5 soil–water suspension using a Leici pH meter. Given the high organic carbon content of coal gangue tailings, the C:N and N:P ratios in this study were calculated using DOC, NH_4_^+^-N, and NO_3_^−^-N, as these forms are most directly relevant to microbial utilization. All measurements were tested three times and the average was recorded as the value.

### 2.3. Heavy Metal Fractionation Analysis

Heavy metals were sequentially extracted into five fractions according to the Tessier method [17]. Exchangeable fraction: 1 g of pretreated soil was mixed with 8 mL of 1 mol L^−1^ MgCl_2_ (pH = 7.0) in a 20 mL or 50 mL centrifuge tube, shaken at 300 rpm at 25 °C for 1 h, centrifuged at 4000 rpm for 10 min, and filtered. The residue was washed with 5 mL of deionized water, and the combined supernatants were diluted to 40 mL for analysis by atomic absorption spectrometry (AAS). Carbonate-bound fraction: The residue from the previous step was mixed with 8 mL of 1 mol L^−1^ sodium acetate (pH = 5.0), shaken at 300 rpm for 5 h at 25 °C, and processed as above. Fe-Mn oxide-bound fraction: The residue was mixed with 20 mL of 40 mmol L^−1^ hydroxylamine hydrochloride (NH_2_OH·HCl in 25% acetic acid), shaken at 300 rpm for 6 h at 96 °C, and processed similarly. Organic matter-bound fraction: The residue was treated with 3 mL of 20 mmol L^−1^ HNO_3_ and 5 mL of 30% H_2_O_2_ (pH = 2.0), and intermittently shaken at 85 °C for 3 h. After cooling, 5 mL of 3.2 mol L^−1^ ammonium acetate (in 20% HNO_3_) was added, diluted to 20 mL with distilled water, and shaken at 300 rpm for 0.5 h before processing. Residual fraction: The final residue was digested with 3 mL concentrated HNO_3_ and 5 mL concentrated HClO_4_ at 96 °C for 18 h and analyzed as described.

### 2.4. Molecular Biological Analysis

Soil samples for molecular analysis were sent to a collaborating company. DNA extraction was performed using the Eppendorf FastDNA Spin Kit, and DNA purity was verified by UV absorbance at 280 nm and 230 nm. Qualified DNA was amplified and sequenced on the Illumina platform, targeting the bacterial V3–V4 region and the fungal ITS2 region [16]. FASTP (version 0.14.1) was applied to carry out quality control on the raw sequence data with a sliding window (-W4-M20). Primer sequences were also eliminated using the CUTADAPT software program. The paired-end clean reads were subsequently merged with the usearch -fastq_mergepairs command, which relies on overlap between paired-end reads. Chimeric sequences were removed through usearch-cluster_otus commands. The merging criteria required a minimum of 16 bp overlap between reads generated from opposite ends of the same DNA fragment, with no more than five mismatches permitted within the overlap region. The merged sequences were designated as raw sequences. FASTP (version 0.14.1) was then employed once more to perform quality control on the raw sequence data using a sliding window (-W4-M20), yielding paired-end clean sequences. Sequences sharing more than 97% nucleotide similarity were clustered into operational taxonomic units (OTUs). The bacterial OTUs were annotated based on the Silva database, whereas the fungal OTUs were annotated based on the UNITE database. The numbers of bacterial reads and fungal reads were evenly extracted to 46,095 and 28,067 in every sample, respectively.

### 2.5. Data Analysis

Statistical analysis was conducted using SPSS v2.0 (Chicago, IL, USA). Differences between groups were assessed by Tukey’s HSD test. Heavy metal composition was analyzed via dimensionality reduction in SPSS, and correlations were evaluated using Pearson’s method. Principal coordinate analysis (PCoA) and LEfSe (linear discriminant analysis effect size) for biomarker identification were performed on the Magigene Company (Guangzhou, China) platform. Microbial interaction networks were constructed using the MENA platform with a cutoff correlation coefficient of 0.89, based on non-log-transformed Spearman correlations and assembled via a maximal greedy approach. Coupling analysis with environmental factors was conducted by incorporating these factors into the microbial network to reconstruct an integrated network that includes both microbial taxa and environmental variables.

## 3. Results

### 3.1. Dynamics of Basic Soil Physicochemical Properties

Both TC and TN exhibited an initial decline followed by a gradual increase along the successional gradient, as they reached the highest levels at S1 (15% and 0.4%, respectively) and their lowest at S4 (4.6% and 0.187%, respectively). However, both remained significantly higher than the average values for typical forest soils in China (maximum ~2% for TC and ~0.15% for TN). This is primarily attributed to the presence of persistent coal gangue and its weathered debris, which contain recalcitrant forms of carbon and nitrogen. Despite potential losses of TC and TN during S1–S4 due to leaching and erosion, the residual coal gangue material maintained its elevated levels. From S4 to S7, both TC and TN increased again, with S7 showing increments of approximately 6% and 0.1% compared to S4.

**Figure 2:**
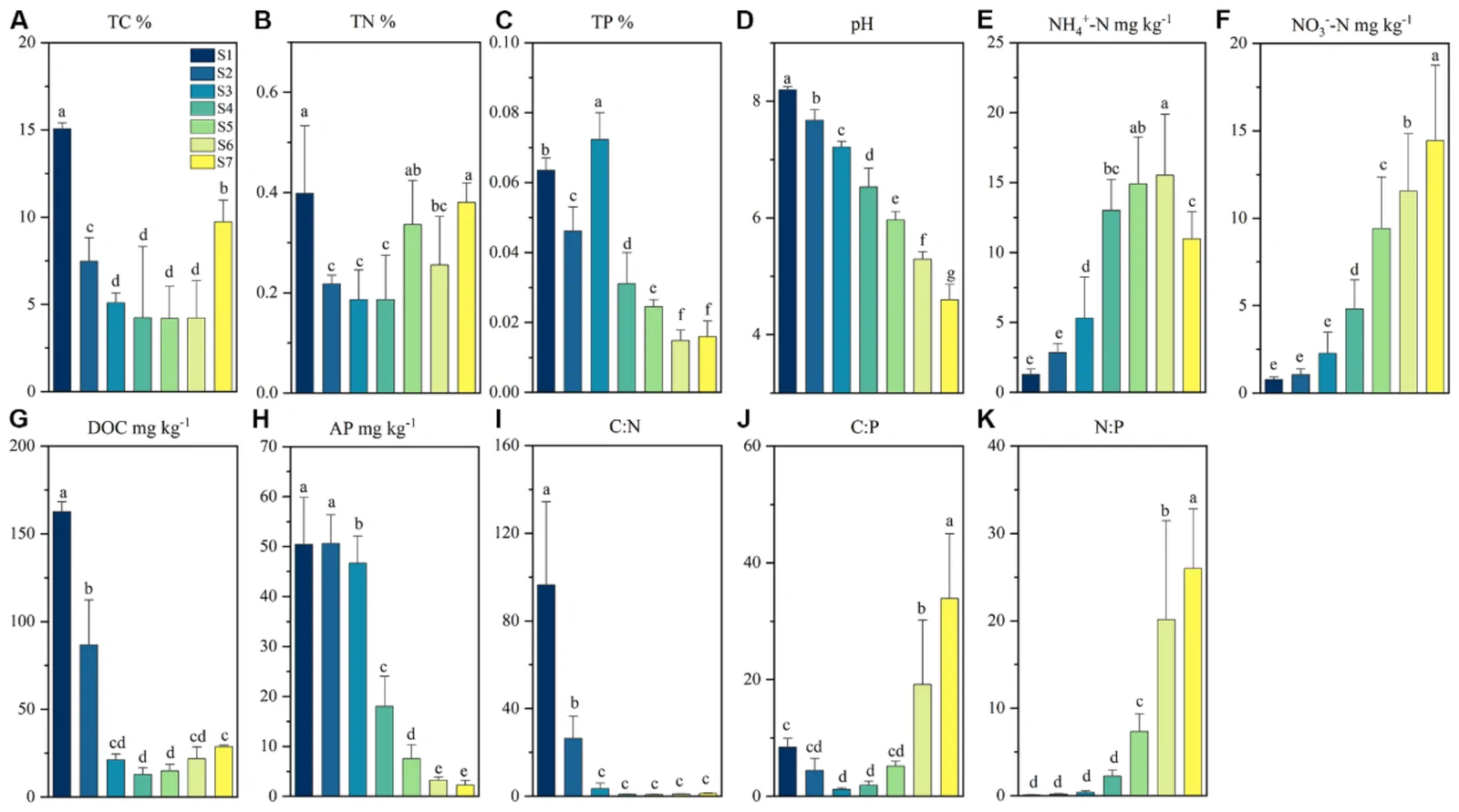
Successional patterns of soil physicochemical properties; the TC, TN, TP, DOC, and AP of the subfigure (**A**–**C**,**G**,**H**) indicate total carbon, total nitrogen, total phosphorus, dissolved organic carbon, and available phosphorus, respectively. The subfigure (**D**–**F**,**I**–**K**) depicts the pH, ammonia nitrogen content, nitrate nitrogen content, the ratio of dissolved organic carbon to the sum of ammonia and nitrate, the ratio of dissolved organic carbon to available phosphorus, and the sum of ammonia and nitrate to available phosphorus. The different lowercase letters in the figure indicate significant differences between groups (*p* < 0.05, Tukey’s HSD, n = 10).

Soil total phosphorus showed a decreasing trend, with the highest value at S3 (0.063%) and the lowest at S6 (0.014%). The levels of TP across the successional stages ranged from moderate to deficient compared to global red soil phosphorus levels (0.03–0.15%), indicating a transition toward phosphorus limitation (Figure 2C). Soil total phosphorus is primarily derived from parent material weathering. Soil acidification following plant colonization further exacerbates phosphorus loss. Soil pH decreased from around 8.2 to 4.6 over 120 years, approaching neutrality by S3. Further acidification occurred with the colonization of fast-growing broadleaf trees and coniferous species (Figure 2D).

Ammonium nitrogen and nitrate nitrogen, being the most readily absorbed nitrogen forms by microorganisms, better represent nitrogen bioavailability than TN. Both NH_4_^+^-N and NO_3_^−^-N generally increased along the successional gradient, although NH_4_^+^-N declined slightly at S7. NH_4_^+^-N rose from 1.3 mg kg^−1^ (S1) to 15.2 mg kg^−1^ (S6), while NO_3_^−^-N increased from 1.1 mg kg^−1^ (S1) to 14.3 mg kg^−1^ (S7). During stages S4–S6, NH_4_^+^-N levels slightly exceeded the average for natural ecosystems (Figure 2E,F).

Dissolved organic carbon followed a trend similar to TC, decreasing initially and then increasing slightly. However, the later increase was much smaller than the earlier decline: DOC decreased by nearly 150 mg kg^−1^ from S1 to S4, but increased by only about 20 mg kg^−1^ from S4 to S7 (Figure 2G).

Available phosphorus declined steadily from 50 mg kg^−1^ (S1) to 2.7 mg kg^−1^ (S7), with a notable decrease beginning at S4, likely due to enhanced phosphate leaching under acidic soil conditions. From S5, AP levels fell below the phosphorus deficiency threshold of 15 mg kg^−1^, typical in red soil regions, indicating increasing phosphorus limitation in the ecosystem (Figure 2H).

Given that TC and TN in early stages largely represent unavailable forms stored within coal gangue, stoichiometric ratios were calculated using DOC, NH_4_^+^-N, NO_3_^−^-N, and AP (though early DOC may also contain less available fractions). These ratios more accurately reflect relative bioavailability constraints. The C:N ratio declined significantly during early succession and stabilized after S4. The C:P ratio initially decreased, reaching a minimum of 1.2 at S3, then gradually increased to 35 by S7. The N:P ratio rose steadily from 0.1 (S1) to 25.4 (S7) (Figure 2I–K).

### 3.2. Patterns of Microbial Community Composition

**Figure 3:**
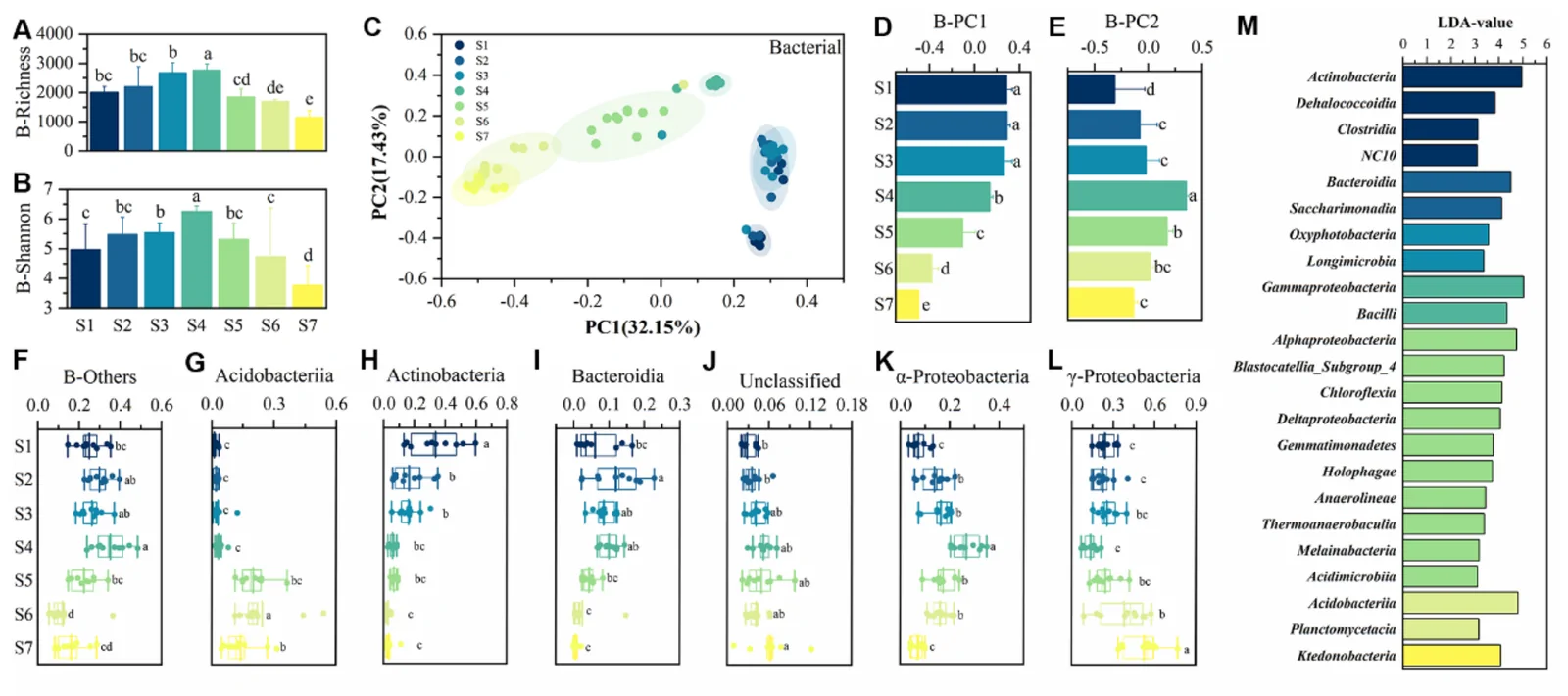
Variations in bacterial community α-diversity and β-diversity. The subfigure (**A**,**B**) depictesthe Shannon index and richness of bacterial community; PC in the subfigures (**C**–**E**) indicates principal axis; the subfigures (**F**–**L**) depictes the variation on bacterial groups on Phylum level; the different letters by the bars in the subfigures indicate significant differences between groups (*p* < 0.05, Tukey’s HSD, n = 10); LDA-value in subfigure (**M**) indicates the value of the linear discriminant analysis.

Bacterial α-diversity indices exhibited an initial increase followed by a decrease. Richness rose from 2000 (S1) to 2800 (S4), then declined to 1100 (S7), while the Shannon index increased from 5 (S1) to 6.4 (S4) before dropping to 3.7 (S7) (Figure 3A,B). PCoA revealed significant shifts in bacterial community structure from S1 to S7, particularly during S4–S7, whereas changes during S1–S3 were less pronounced (Figure 3C). Statistical analysis of PC1 and PC2 indicated no significant variation in PC1 across S1–S3, though PC2 differed significantly between S1 and S2 (Figure 3D,E).

At the class level, the “Others” category (representing rare taxa) did not change significantly during S1–S3 but differed markedly at S6 and S7 compared to earlier stages (Figure 3F). *Acidobacteriia* remained low (<5%) before S4, when soil acidification was not yet pronounced, but increased significantly to over 15% after S5 (Figure 3G). In contrast, *Actinobacteria* gradually declined from 32% to nearly 0 (Figure 3H), and *Bacteroidia* decreased from 6% to near disappearance (Figure 3I). Unclassified taxa increased slightly from 0.03% to 0.06% (Figure 3J). α-*Proteobacteria* showed a hump-shaped trend, rising from 6% (S1) to 24% (S4), then falling to 5% (S7) (Figure 3K), while γ-*Proteobacteria* remained at around 20% during S1–S3 but increased from S4 to reach over 50% by S7 (Figure 3L). Using LEfSe analysis (LDA > 3), biomarker bacterial genera were identified for each stage: S4 had the most (10 genera), followed by S1 (four genera), S2, S3, and S6 (two each), and S7 (one genus) (Figure 3M).

**Figure 4:**
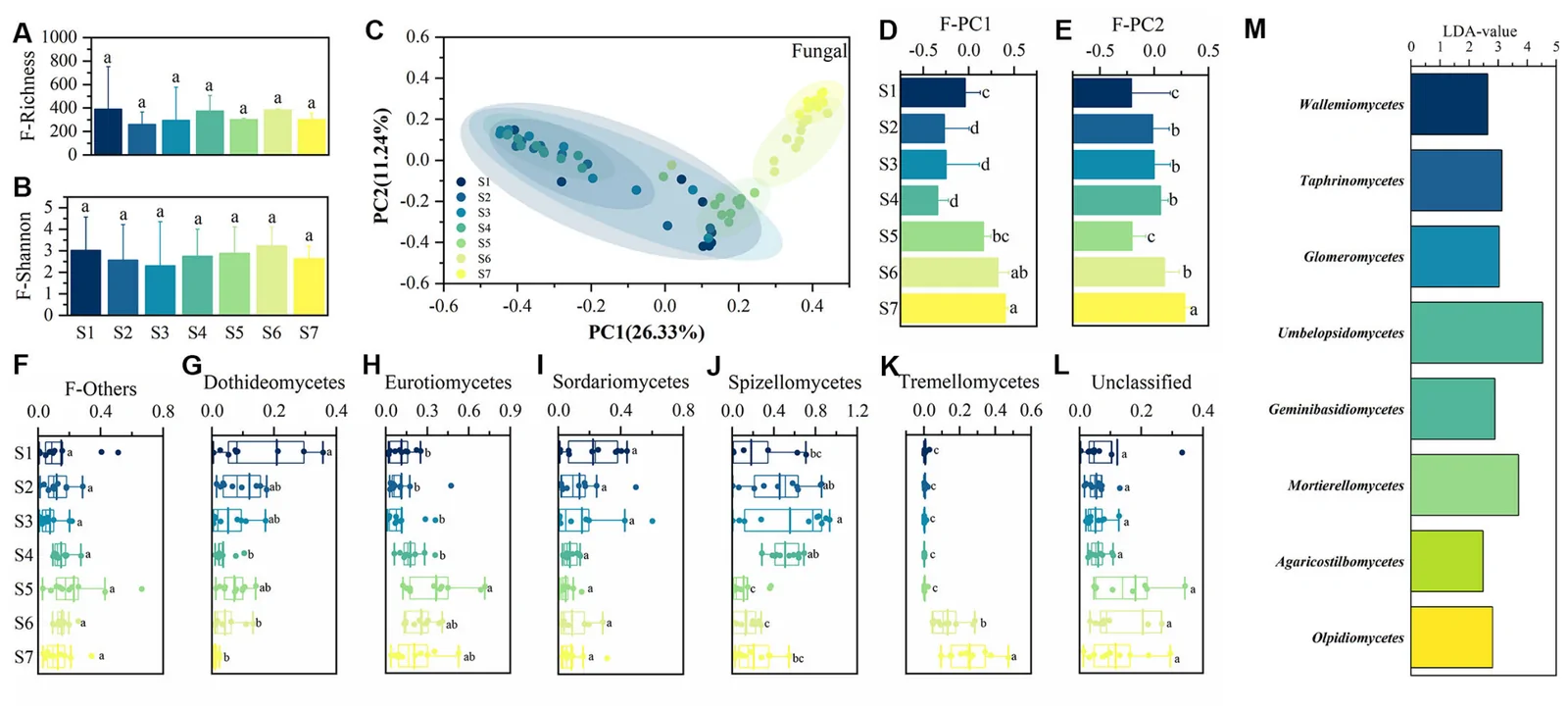
Variations on fungal community α-diversity and β-diversity. The subfigure (**A**,**B**) depictesthe Shannon index and richness of fungal community; PC in the subfigures (**C**–**E**) indicates principal axis; the subfigures (**F**–**L**) depictes the variation on fungal groups on Class level; the different letters by the bars in the subfigures indicate significant differences between groups (*p* < 0.05, Tukey’s HSD, n = 10); the LDA-value in subfigure (**M**) indicates the value of linear discriminant analysis.

Fungal α-diversity did not change significantly across succession. Richness ranged from 227 (S2) to 389 (S1), and the Shannon index varied between 2.47 (S3) and 3.12 (S6) (Figure 4A,B). PCoA indicated significant overall shifts in fungal community structure from S1 to S7 (Figure 4C). PC1 and PC2 differed significantly between S1 and S2 but remained stable from S2 to S4, suggesting that initial plant colonization strongly shapes fungal communities, with limited further change during the grassland phase. After S4, both PC1 and PC2 increased markedly, indicating continued community restructuring with woody plant establishment (Figure 4D,E).

The relative abundance of fungal “others” showed no consistent trend and varied considerably, reflecting stochastic assembly processes during succession (Figure 4F). *Dothideomycetes* were highest at S1 (~20%) and significantly lower thereafter (~10% during S2–S6), possibly influenced by plant-derived organic matter (Figure 4G). *Eurotiomycetes* remained at 10–15% during S2–S4 but increased sharply to 40% at S5 (Figure 4H). *Sordariomycetes*, though relatively high at S1 (~20%), showed high within-group variability and no significant between-stage differences (Figure 4I). *Spizellomycetes* peaked at S3 (~50%) before declining to 15–25% during S5–S7 (Figure 4J). *Tremellomycetes* were nearly absent during S1–S5 but increased to 17.5% and 25% at S6 and S7, respectively, with coniferous forest development (Figure 4K). Unclassified fungal taxa increased after tree colonization (S4) but without significant between-stage differences (Figure 4L). Using LEfSe (LDA > 2), biomarker fungal genera were most abundant by S4 (three genera), followed by S4 (two genera), with other stagesfollowed by S5 to S7, those having only 1 genus each (Figure 4M).

The detailed variation in bacterial and fungal community composition by Class, Order, Family and Genus level is listed in Figures S1 and S2. 

### 3.3. Dynamics of Heavy Metal Fractionation

Across the successional gradient, exchangeable (A) and carbonate-bound (B) fractions of all five metals generally peaked at the initial stage (S1) and declined markedly in later stages, indicating reduced metal mobility with succession. By contrast, the residual fraction (E) exhibited consistent increases, particularly for As, Cr and Mn, suggesting progressive metal stabilization into geochemically stable forms. For Ni, Zn and Cd, Fe-Mn oxide-bound (C) and organic-bound (D) fractions displayed V-shaped trends: decreasing to minima at mid-succession (S3–S5) and rebounding in late succession (S6–S7). Notably, organic-bound and residual Ni both reached maximum values at S7. Arsenic showed unique patterns: exchangeable and carbonate-bound As decreased continuously, while Fe-Mn oxide-bound, organic-bound and residual As increased toward late succession, with residual As increasing ~4-fold from S1 to S6. Chromium differed from other metals: exchangeable and carbonate-bound Cr decreased only slightly after S1, whereas Fe-Mn oxide-bound, organic-bound and residual Cr increased gradually, confirming steady Cr immobilization throughout succession (Figure S1).

**Figure 5:**
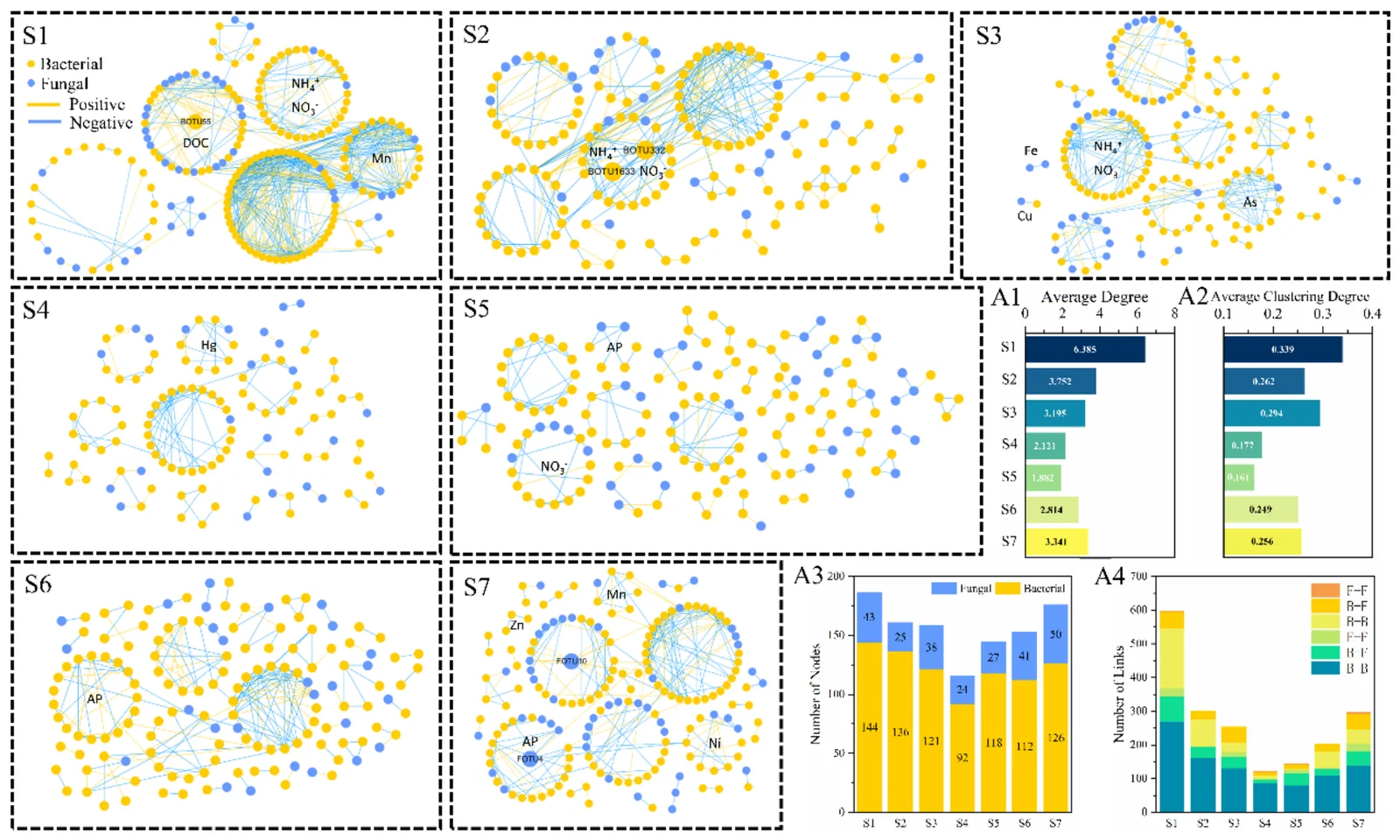
Topological properties of cross-domain networks across stages. In the subfigures (**S1**–**S7**), the yellow dots, blue dots, yellow lines and blue lines indicate bacterial OTUs, fungal OTUs, positive interactions and negative interactions, respectively. The subfigure (**A1**–**A3**) depicts the average degree, the average clustering degree and the number of network nodes, respectively. The F and B in subfigure (**A4**) indicate fungal and bacterial, respectively.

Copper, Fe and Hg displayed moderate shifts in fraction distribution, with overall weaker successional responses than the metals in Figure 5. Exchangeable Cu increased to a mid-succession peak before declining, while residual Cu decreased initially then recovered. Iron showed strong redistribution: organic-bound Fe fell sharply to a minimum at S3 then rose to the highest level at S7, while residual Fe peaked at mid-succession. Mercury fractions remained relatively stable across stages, with only small fluctuations in Fe-Mn oxide-bound and organic-bound Hg. Manganese exhibited the strongest and most consistent successional enrichment: Fe-Mn oxide-bound, organic-bound and residual Mn all increased continuously, reaching maxima at S6 with 2–13-fold increases relative to S1. Lead showed moderate mobility reduction: carbonate-bound Pb decreased steadily, while residual Pb increased gradually toward late succession, accompanied by fluctuating organic-bound Pb (Figure S2).

Most heavy metal fractions followed the general order residual (E) > organic-bound (D) > Fe-Mn oxide-bound (C) > carbonate-bound (B) > exchangeable (A). However, for Fe and Mn, the organic-bound fraction (D) exceeded the residual fraction (E), which may be attributed to their higher prevalence in organic-associated forms within the coal gangue matrix.

The detailed data for the successional trajectory of heavy metal fractions are listed in Figure S2. The covariation of heavy metal fractions were comprehensively described in Figure S3.

### 3.4. Successional Dynamics of Microbial Network Structure

Contrary to α-diversity trends, microbial interaction networks showed an initial simplification followed by increasing complexity along the successional gradient. Average connectivity and the average clustering coefficient decreased from S1 to S5, then increased from S5 to S7 (Figure 5(A1,A2)). Total node counts were lowest at S4 (~120), compared to nearly 180 at S1 and S7. Total connections dropped sharply from ~600 at S1 to ~130 at S4, then recovered to ~300 at S7 (Figure 5(A3,A4)). Network topology visually reflected these shifts.

At S1, the network exhibited the most complex interactions and clear modular specialization. In addition to the largest module, three major modules were associated with DOC, inorganic nitrogen (NH_4_^+^-N and NO_3_^−^-N), and Mn, with DOC directly linked to keystone taxon BOTU55, suggesting early network organization around carbon, nitrogen, and manganese availability. By S2, network size remained similar but connection density halved. Two connector taxa (BOTU1633 and BOTU332) emerged, linking different functional modules and likely enhancing functional integration. Their close associations with NH_4_^+^-N and NO_3_^−^-N indicated a central role of nitrogen cycling during early plant colonization. At S3, network size persisted but connectivity further declined, with reduced coupling between modules. The central module remained structured around inorganic nitrogen. S4 had the smallest and least connected network, with only two inter-module links. Except for Hg, no nutrients or heavy metals were associated with modules, suggesting a transitional or functionally decoupled state. At S5, networks became even simpler, with complete loss of inter-module connections and increased isolation of small modules. NO_3_^−^-N appeared in the central module, and AP emerged in peripheral modules, marking the initial influence of phosphorus availability. By S6, network complexity recovered, and the central module became increasingly organized around AP. At S7, several large modules containing keystone taxa (e.g., FOTU4) reappeared, with both within- and between-module interactions partially restored. One core module was directly linked to AP, while Ni, Mn, and Zn were associated with smaller modules. Overall, network assembly shifted from focusing on carbon and nitrogen to emphasizing phosphorus limitation, with certain heavy metals shaping network structure at specific mediate stages.

### 3.5. Analysis of Driving Factors Underlying Community Succession Dynamics

The heavy metal fractions were extracted into several factors, and the two factors with the strongest explanatory power, denoted as HPC1 and HPC2, were used for correlation analysis (Table S1). The factors most strongly correlated with FPC1 were AP and pH, while FPC2 was most closely associated with pH and HPC2. For BPC1, the highest correlations were observed with pH, AP, and AN. BPC2 showed the strongest correlation with DOC. Bacterial richness was most influenced by pH, whereas fungal richness was primarily linked to HPC2. These results suggest that heavy metals may inhibit rare taxa, thereby reducing both fungal and bacterial richness, with HPC2-represented metal elements playing a key role (Table 1).

The average connectivity (avgK) of the microbial network was most correlated with DOC and HPC1, indicating that DOC availability and HPC1-associated metals can regulate network interaction density by influencing energy and carbon accessibility. Higher heavy metal concentrations may drive microbial communities to enhance interactions, such as carbon exchange and shared extracellular secretions, to mitigate metal toxicity, thereby increasing connection density. Nevertheless, nutrient availability and carbon sources emerged as the most critical factors shaping community composition, consistent with earlier network analyses. Although heavy metals also contributed to structuring microbial assemblages, their influence was secondary to that of nutrient dynamics (Table 1).

## 4. Discussion

### 4.1. Temporal Evolution Characteristics and Driving Mechanisms of Heavy Metal Fractions in Soils of Abandoned Coal-Mining Sites in Southern China

Our results demonstrate that the proportion of acid-extractable heavy metal fractions generally declined in coal mine soils with succession time, while oxidizable and residual fractions gradually increased, and the reducible fraction fluctuated. Heavy metals were progressively transformed from highly active fractions into more stable bound forms. Such fraction-evolution patterns are consistent with findings from existing studies on coal-mining and black shale-mining areas [18,19]. Soils from black shale-mining sites in Hunan, China, revealed that Cd mainly occurred in the acid-soluble phase; nevertheless, progressive weakening of sulfide oxidation over weathering time drove partial transformation of heavy metals toward the residual phase [18]. A synthetic analysis of soils from 50 coal mines across China also indicated that although total concentrations of Cd, Pb, and Ni in coal mine soils exceeded regional background values, substantial fractions of these heavy metals were immobilized within mineral lattices and organic-matter-bound phases, with active fractions decreasing over time [19]. Mechanistically, slight soil pH recovery, accumulation of soil organic matter, and formation of Fe-Mn oxides after mine abandonment constitute the core factors promoting heavy metal passivation [20]. On the one hand, acid-producing sulfide-mineral oxidation gradually subsides following mine abandonment, soil acidity is partially buffered, lattice destruction induced by hydrogen ions is mitigated, and thus heavy metal dissolution is reduced. On the other hand, plant litter and root exudate input during ecological succession elevate soil organic-matter contents. Functional groups of organic matter convert acid-extractable heavy metals into oxidizable fractions via complexation and adsorption. Meanwhile, Fe-Mn (hydr)oxides generated by weathering trap heavy metals through co-precipitation and surface adsorption, raising the proportions of residual and reducible fractions [21].

Nevertheless, certain elements (e.g., Cd) still maintained considerable proportions of acid-extractable fractions in the present study, which differs from observations in some metal-mining sites. Surveys of numerous Pb-Zn and tungsten–molybdenum polymetallic mines have shown that residual fractions dominate heavy metal speciation in late-stage abandoned mines, with negligible proportions of active fractions [20]. Three potential explanations account for this discrepancy. First, parent-rock mineralogical compositions differ greatly between coal mines and polymetallic mines. Fine-grained associated sulfides (sphalerite, pyrite) persist in coal mine substrates; even after mining ceases, these residual fine sulfides undergo slow oxidation and continuously release bioavailable heavy metals [18]. By contrast, polymetallic tailings lose most readily weatherable sulfides after mineral-processing beneficiation, leaving behind more stable residual minerals. Second, southern China features high temperature and abundant rainfall; rainfall leaching coupled with alternating wet–dry conditions repeatedly disturbs soil redox conditions and impedes complete conversion of heavy metals into residual fractions, so a portion of heavy metals remains in active acid-extractable forms over the long term [22]. Third, our study investigated in situ natural succession of abandoned coal mines without artificial soil-covering or passivation remediation, whereas many metal mine studies focus on anthropogenically remediated sites with higher degrees of heavy metal passivation [22,23].

In addition, total heavy metal concentrations and fraction variations were not fully synchronized. In this work, total contents of some heavy metals showed no significant decline, yet the percentages of their active fractions decreased markedly. This observation agrees with a national meta-analysis, which reported that the total heavy metal concentrations in mining-area soils are largely controlled by the parent-rock background, whereas bioavailability is governed by heavy metal fractions rather than total concentrations [24]. This implies that ecological risk assessments for southern coal mine areas relying merely on total heavy metal contents would overestimate actual ecological hazards; fraction-based indices are therefore indispensable [25]. Even with reduced active-fraction proportions, Cd still retained notable acid-extractable pools, which carry potential risks of remobilization under disturbances such as secondary acid drainage triggered by intense rainfall. This issue merits attention for long-term ecological management of abandoned coal-mining sites in southern China [26].

### 4.2. Nutrients Exert Stronger Control over Soil Microbial Community Succession than Heavy Metals in Coal-Mining Areas

Results of variance partitioning and redundancy analysis indicate that soil nutrients (soil organic matter, total nitrogen, available phosphorus, etc.) explain significantly more variance in microbial community composition, diversity and succession trajectories than either total or bioavailable heavy metal concentrations. During in situ succession at abandoned coal mines in southern China, nutrient limitation serves as the primary stressor shaping microbial community succession, while the selective pressure imposed by heavy metals plays a secondary role [27,28].

These findings are supported by multiple restoration-focused mining-area investigations. One study focusing on abandoned coal-mining sites in central China reported that despite heavy metal concentrations above regional background levels, soil organic carbon and nitrogen supply represented the foremost environmental factors explaining bacterial community variation, with low independent explanatory power from heavy metals [28]. Research on reclaimed coal-subsidence soils further confirmed that under oligotrophic substrate conditions; carbon–nitrogen nutrient supply determines the magnitude of microbial diversity improvement [27]. Heavy metals only filter a small subset of tolerant taxa and cannot drive whole-community temporal succession. Underlying mechanisms can be summarized into three aspects. Firstly, the severe inherent nutrient deficiency of substrates: abandoned coal mine substrates contain intrinsically low quantities of organic matter and limited nitrogen and phosphorus pools. In the early stages of succession, microorganisms primarily suffer from carbon and nitrogen resource starvation; resource limitation masks the toxic effects of heavy metals. Microbes prioritize acquiring energy and nutrients, and heavy metal toxicity only manifests when nutrient availability is sufficient [29]. Secondly, the development of heavy metal-resistant microbial reservoirs via long-term site acclimatization: long-term mining disturbances enrich heavy metal-tolerant taxa (e.g., *Proteobacteria* or *Actinobacteriota*) in soils. The overall community acquires heavy metal resistance, weakening community-wide selective filtering by heavy metals, and resource competition becomes the major driver of community turnover [27]. Thirdly, reduced actual biological toxicity originating from heavy metal speciation: consistent with our fraction data, large quantities of heavy metals are transformed into residual and oxidizable fractions over succession. Only limited proportions exist as acid-extractable forms that are biologically accessible to microbial cells. Bioavailable heavy metal concentrations never reach thresholds for strong community-scale inhibition; total heavy metal contents overestimate real microbial exposure risks [19].

Still, our conclusions diverge from some studies conducted on tailings and smelter-contaminated sites, where heavy metals are reported as the dominant factor governing microbial community variation [30]. Three reasons may explain such inconsistencies. First, pollution load differences: smelter and Pb-Zn tailing sites exhibit far higher bioavailable heavy metal concentrations than the coal mine soils examined here. Extremely high bioavailable heavy metal levels become overriding stressors, whereas heavy metal pools in coal mine soils mainly stem from parent-rock weathering with limited bioactive fractions [20]. Second, soil pH discrepancies: strongly acidic tailing sites maintain low pH values that promote heavy metal dissolution and amplify toxicity. By comparison, soils in our late-succession sites experienced partial pH buffering that lowered heavy-metal bioavailability [22]. Third, differences in ecological succession stages: heavy metal-induced transient stress may prevail at the very initial stage immediately after coal-substrate exposure. Our sampling covered mid-to-late succession stages; after prior community acclimatization, nutrient constraints became more prominent [31].

We do not negate the influences of heavy metals in this system. Although not the primary succession driver, heavy metals still significantly alter the relative abundances of specific microbial taxa and reconfigure microbial co-occurrence-network modules. Bioavailable fractions of Cd and Hg exert inhibitory effects on certain sensitive fungal groups. Accordingly, heavy metal risks cannot be completely disregarded for coal mine ecological restoration; nevertheless, nutrient scarcity constitutes the bottleneck constraining microbial community recovery [32]. For practical in situ restoration of southern abandoned coal mines, our results suggest that prioritizing soil carbon–nitrogen–phosphorus improvement is more conducive to rebuilding soil microbial ecosystems than heavy metal passivation alone.

### 4.3. Research Limitations and Future Perspectives

This study adopted the space-for-time substitution approach to reveal that nutrients exert a more dominant driving effect than heavy metals on soil microbial community succession at abandoned coal-mining sites in southern China. Nevertheless, this method is susceptible to confounding heterogeneity from parent-rock materials and micro-topography across sampling plots, and field observational data alone cannot provide direct evidence verifying the dominant role of nutrients [33,34]. Future controlled nutrient-addition simulation experiments should be performed to validate nutrient-mediated regulatory effects. Combined with multiple technical approaches including micro-mineralogical characterization, meta-omics, rhizosphere micro-zone analysis and biological toxicity assays, we should disentangle the interactive coupling mechanisms among nutrients, heavy metals and microorganisms. Building upon mechanistic insights derived from basic research, practically applicable auxiliary-remediation technical systems for mining sites can be established to improve ecological-reconstruction efficiency and accelerate natural-restoration processes for abandoned coal-mining sites across southern China [6].

During the review, we noticed that the present study attributes soil acidification mainly to plant and microbial metabolism. Nevertheless, acid mine drainage from minerals can also serve as a critical driver of soil acidification and affect the mobility, concentration, and toxicity of heavy metals in soil. Accordingly, disentangling biotic and abiotic processes over succession, alongside quantifying variations in their relative contributions to environmental consequences, constitutes a promising research avenue for future studies on accelerated natural restoration [35].

## 5. Conclusions

Through an investigation of microbial communities and environmental factors along a century-old coal gangue successional gradient in southern China, we observed a transition in the mine soil from alkaline to acidic conditions and a shift from carbon and nitrogen limitation to phosphorus limitation. Heavy metal fractions exhibited complex successional trends: most showed either increasing or decreasing tendencies over time, some displayed initial decreases followed by increases (or vice versa), and a minority were specifically enriched or depleted during the broadleaf forest stages, alongside other distinct patterns. The cross-domain microbial network structure transitioned from complex to simplified and then re-complexified, a dynamic primarily driven by dissolved organic carbon (DOC) and a few heavy metal components. However, the shift in nutrient limitation—from carbon and nitrogen scarcity to phosphorus scarcity—emerged as the most critical factor shaping the assembly of core functional modules and determining community composition. Therefore, we propose that in early successional stages, the application of organic amendments and nitrogen fertilizers should be prioritized to accelerate microbial community development toward later stages. In contrast, during later stages, phosphorus fertilization is essential to ensure forest ecosystem quality and stability. It is noteworthy that under natural restoration, most heavy metals tend to accumulate in stable forms such as residual fractions, resulting in low ecological toxicity. Nevertheless, attention should be paid to the increase in water-soluble heavy metals during the broadleaf forest phase, where the application of chelating agents could be considered to mitigate potential toxicity risks.

## Figures and Tables

**Table 1:** Spearman correlations between environmental factors and microbial community properties.

	FPC1	FPC2	BPC1	BPC2	Fungal Richness	Bacterial Richness	avgK
HPC1	−0.371 **	−0.067	0.636 **	−0.610 **	−0.102	0.16	0.905 **
HPC2	0.624 **	0.430 **	−0.612 **	−0.381 **	0.254 *	−0.505 **	0.165
pH	−0.707 **	−0.510 **	0.894 **	−0.166	−0.086	0.517 **	0.530 **
DOC	0.009	0.031	0.308 **	−0.767 **	−0.13	−0.243 *	0.921 **
AN	0.641 **	0.375 **	−0.837 **	0.290 **	0.111	−0.434 **	−0.631 **
AP	−0.740 **	−0.426 **	0.875 **	−0.127	−0.133	0.553 **	0.477 **

Note: * and ** indicate significant correlations at *p* < 0.05 and *p* < 0.01 levels (n = 70), respectively. HPC, FPC, and BPC represent the principal components of heavy metal fractions, fungal communities, and bacterial communities, respectively. DOC, AP and AN indicate dissolved organic carbon, available phosphorus and available nitrogen, respectively.

## Data Availability

The original sequencing data have been uploaded to NCIB with NO. PRJNA1515385 and NO. PRJNA1515439 for bacterial and fungal sequences, respectively.

## Supplementary Materials

The following supporting information can be downloaded at: https://www.mdpi.com/article/10.3390/microorganisms14102322/s1, Figure S1: The subfigure A to D depicts the successional patterns of bacterial community group relative abundances on Class, Order, Family and Genus level, respectively; Figure S2: The subfigure A to D depicts the successional patterns of fungal community group relative abundances on Class, Order, Family and Genus level, respectively; Figure S3: Successional patterns of Ni, Zn, As, Cd and Cr fractions. A–E sequentially represent exchangeable (A), carbonate bound (B), Fe Mn oxide bound (C), organic matter bound (D), and residual (E) fractions. The different letters upon the bars in subfigures indicates significant differences between groups (*p* < 0.05, Tukey’s HSD, n = 10); Figure S4: Successional patterns of Cu, Fe, Hg, Mn and Pb fractions. A–E sequentially represent exchangeable (A), carbonate bound (B), Fe Mn oxide bound (C), organic matter bound (D), and residual (E) fractions. The different letters upon the bars in subfigures indicates significant differences between groups (*p* < 0.05, Tukey’s HSD, n = 10); Table S1: Dimensionality reduction factors of heavy metal fractions.
