# Supplementary material for: Soil Nutrients Rather than Heavy Metals Dominate the Succession of Microbial Communities in Subtropical Coal Gangue Piles: A Case Study from a Successional Gradient in Southern China
Source: Microorganisms. 2026 Oct 12;14(10):2322. doi: 10.3390/microorganisms14102322 (PMC13707978; doi:10.3390/microorganisms14102322)
Supplement: Supplementary file 1 [file microorganisms-14-02322-s001.zip › microorganisms-4545065-supplementary.pdf]

Supplementary 1

The variation on bacterial and fungal communities about Class, Order, Family and Genus level.

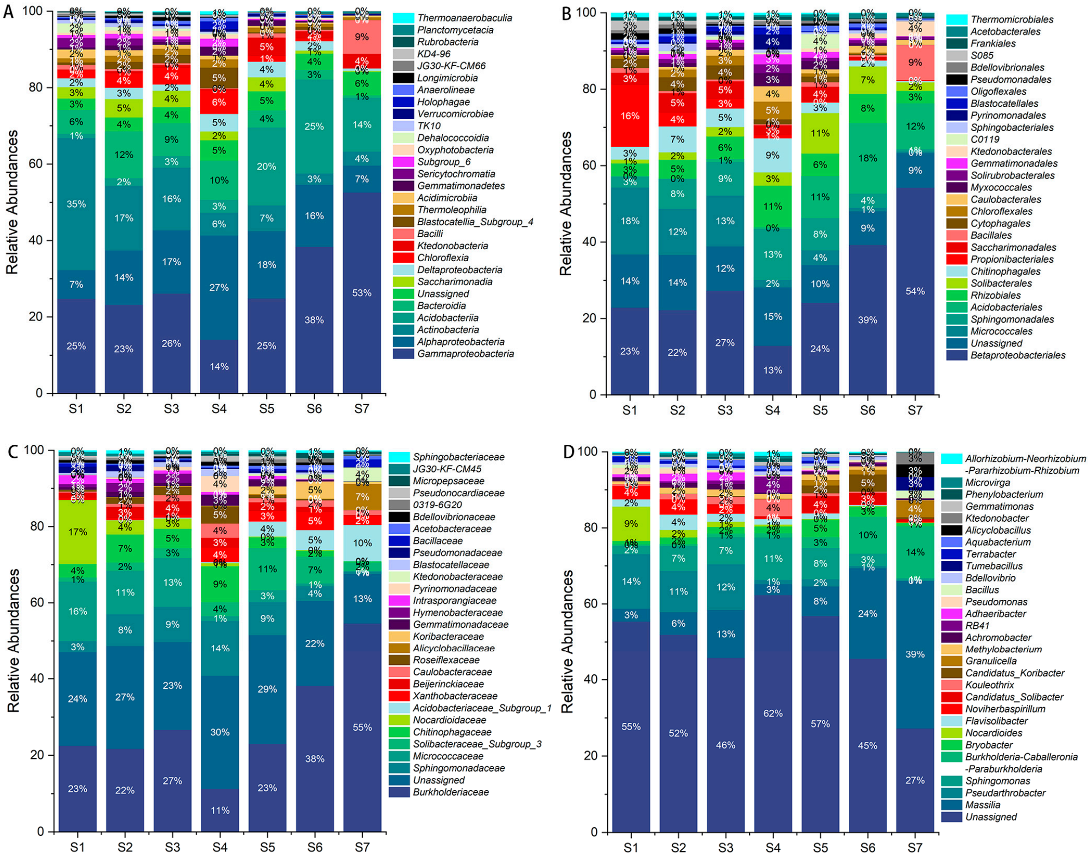

Figure S1: The subfigure A to D depicts the successional patterns of bacterial community group relative abundances on Class, Order, Family and Genus level, respectively.

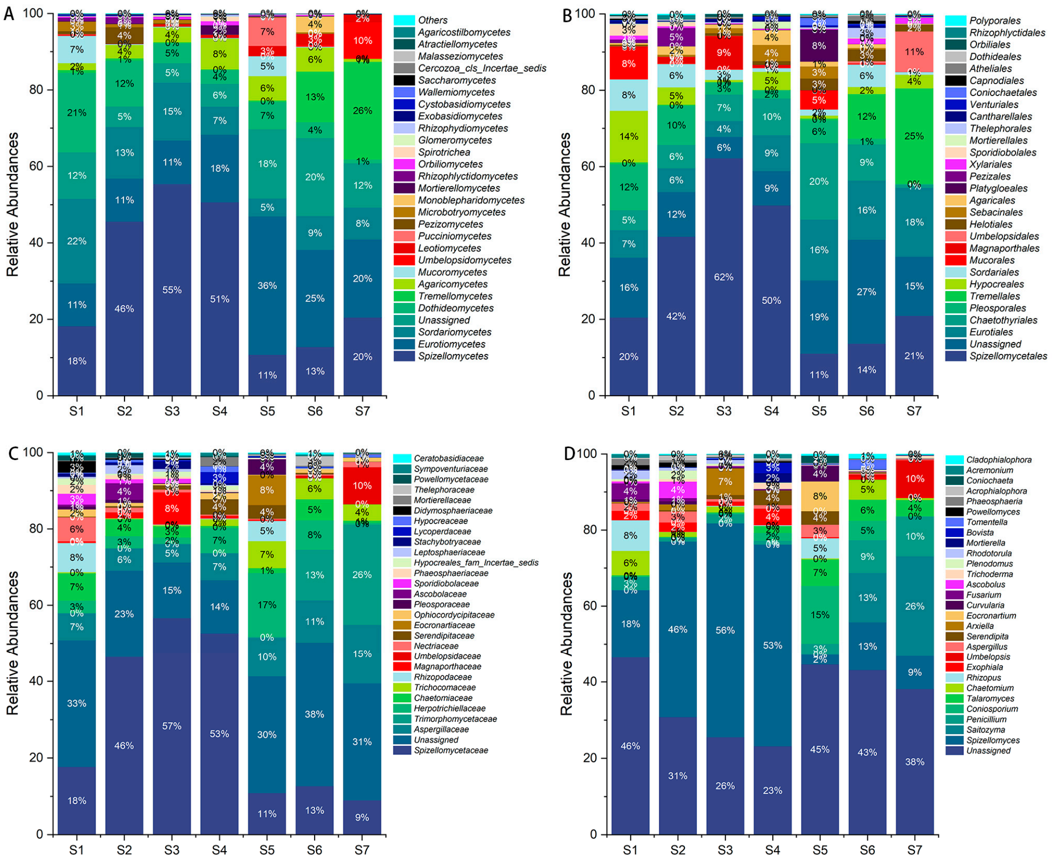

Figure S2: The subfigure A to D depicts the successional patterns of fungal community group relative abundances on Class, Order, Family and Genus level, respectively.

Supplementary 2

Ni-A (exchangeable) initially decreased and then increased along the successional gradient, ranging from 0.22 mg kg^{-1} (S1) to 0.05–0.08 mg kg^{-1} (S2–S6), before rising to 0.37 mg kg^{-1} at S7. Ni-B (carbonate-bound) was significantly higher in S1 and S2 (~0.38 mg kg^{-1}) compared to other stages, declining to 0.135 mg kg^{-1} at S3 and further to 0.044 mg kg^{-1} by S7. Ni-C (Fe-Mn oxide-bound) decreased from 2.87 mg kg^{-1} (S1) to 0.54 mg kg^{-1} (S5), then increased to 3.66 mg kg^{-1} (S7). Ni-D (organic-bound) peaked at S7 (11.2 mg kg^{-1}), with the lowest value at S5 (0.14 mg kg^{-1}); other stages showed no significant differences (0.25–0.45 mg kg^{-1}). Ni-E (residual) followed a similar trend to Ni-D, with the highest value at S7 (43.25 mg kg^{-1}) and the lowest at S5 (22.55 mg kg^{-1}); S1–S4 remained around 30 mg kg^{-1} without significant variation.

Zn-A (exchangeable) increased initially and then decreased, peaking at 25.13 mg kg^{-1} (S5) before declining to 4.67 mg kg^{-1} (S7). S1–S3 had consistently low Zn-A (< 2 mg kg^{-1}) with no significant differences. Zn-B (carbonate-bound) dropped sharply from 6.37 mg kg^{-1} (S1) to 2.24 mg kg^{-1} (S2), remaining below 0.3 mg kg^{-1} from S3 onward. Zn-C (Fe-Mn oxide-bound)

decreased from 15.09 mg kg^{-1} (S1) to 5.01 mg kg^{-1} (S2), reached a minimum at S5 (0.21 mg kg^{-1}), and then increased to 10.23 mg kg^{-1} (S7). Zn-D (organic-bound) was approximately 30 mg kg^{-1} at S5 and S7, but around 60 mg kg^{-1} in other stages. Zn-E (residual) exceeded 400 mg kg^{-1} at S1, decreased to 200–250 mg kg^{-1} during S2–S5, and rose again to ~300 mg kg^{-1} at S6 and S7.

As-A (exchangeable) was highest at S1 (0.53 mg kg^{-1}), with other stages averaging about half this value and the lowest at S4 (0.203 mg kg^{-1}). As-B (carbonate-bound) declined from 0.97 mg kg^{-1} (S1) to 0.14 mg kg^{-1} (S4), followed by a slight but insignificant increase. As-C (Fe-Mn oxide-bound) was highest at S6 and S7 (~4 mg kg^{-1}), while S2 and S5 were around 1 mg kg^{-1}. As-D (organic-bound) peaked at S6 (13.34 mg kg^{-1}), followed by S7 and S1, with the lowest at S2 (4.69 mg kg^{-1}). As-E (residual) was highest at S6 (221 mg kg^{-1}) and lowest at S1 (51.3 mg kg^{-1}).

Cd-A (exchangeable) decreased initially and then increased, declining from 5.82 μg kg^{-1} (S1) to 1.74 μg kg^{-1} (S5) before rising to 3.61 μg kg^{-1} (S7). Cd-B (carbonate-bound) generally decreased from 16.89 μg kg^{-1} (S1) to 2.31 μg kg^{-1} (S7), despite a slight increase at S6. Cd-C (Fe-Mn oxide-bound) was significantly higher at S2 (36.8 μg kg^{-1}) and S3 (24.5 μg kg^{-1}) compared to other stages (2.4–7.3 μg kg^{-1}). Cd-D (organic-bound) initially decreased from 26.5 μg kg^{-1} (S1) to 3.52 μg kg^{-1} (S4), increased to 13.1 μg kg^{-1} (S6), and then dropped sharply at S7 to levels similar to S4. Cd-E (residual) declined overall from 1.69 mg kg^{-1} (S1) to 0.18 mg kg^{-1} (S7).

Cr-A (exchangeable) was significantly higher at S1 (2.92 mg kg^{-1}) than in other stages, decreasing to 2.46 mg kg^{-1} (S2) before gradually increasing again. Cr-B (carbonate-bound) showed a similar trend, with S2 values about three-quarters of those at S1, followed by a slight increase in later stages. Cr-C (Fe-Mn oxide-bound) increased gradually from 4.76 mg kg^{-1} (S1) to over 4.84 mg kg^{-1} (S6). Cr-D (organic-bound) declined overall, with S1–S3 slightly above 9 mg kg^{-1}, dropping sharply at S4, and remaining around 6 mg kg^{-1} thereafter. Cr-E (residual) increased from ~0.22 g kg^{-1} (S1–S2) to ~0.24 g kg^{-1} after S3, peaking at 0.263 g kg^{-1} (S6).

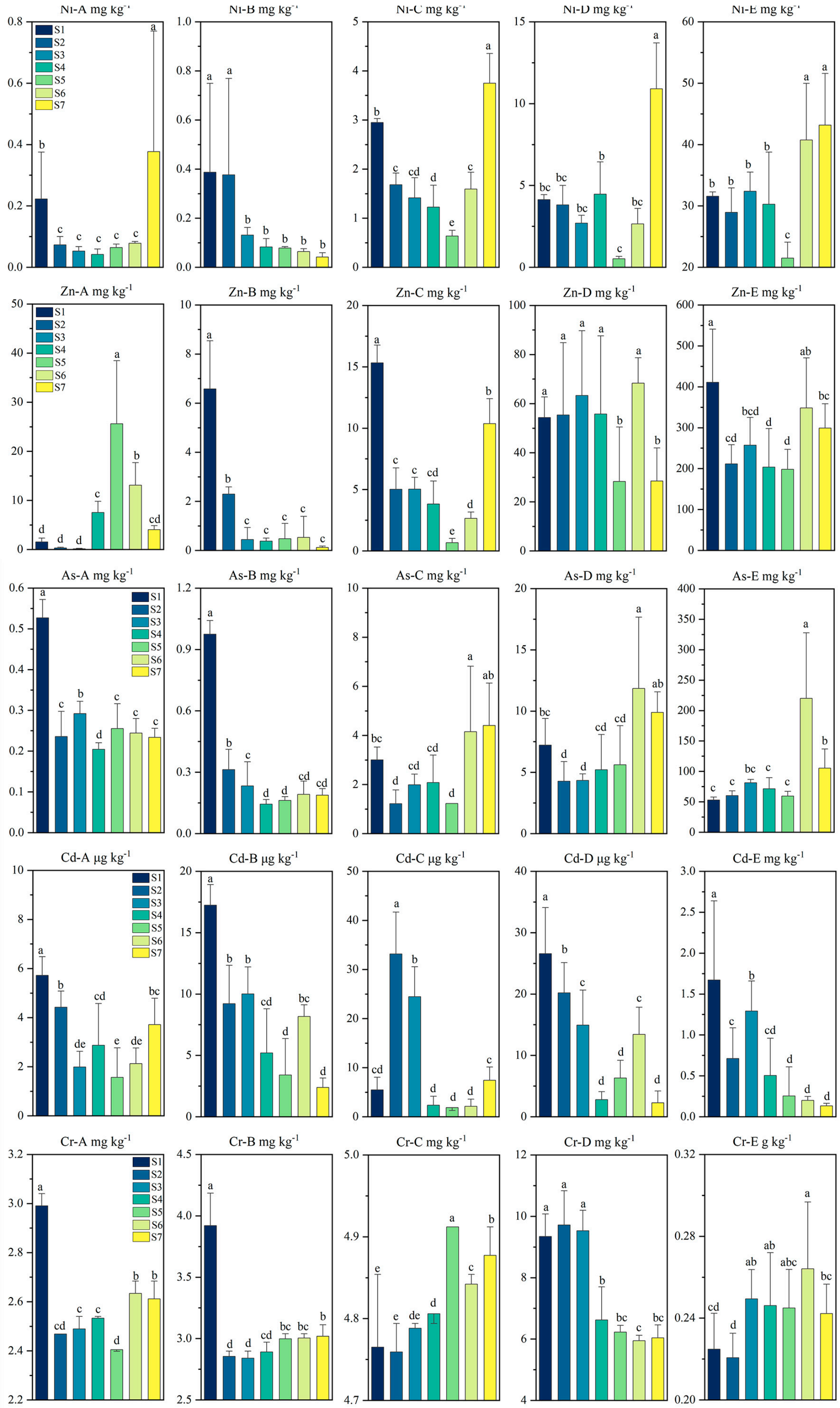

Figure S3: Successional patterns of Ni, Zn, As, Cd and Cr fractions. A–E sequentially represent exchangeable (A), carbonate-bound (B), Fe-Mn oxide-bound (C), organic matter-bound (D), and residual (E) fractions. The different letters upon the bars in subfigures indicates significant differences between groups ($P < 0.05$, Tukey's HSD, n=10).

Cu-A (exchangeable) increased from 2.18 mg kg^{-1} (S1) to 3.22 mg kg^{-1} (S5), then decreased to 2.47 mg kg^{-1} (S7). Cu-B

(carbonate-bound) declined overall from 3.3 mg kg^{-1} (S1) to 2.03 mg kg^{-1} (S7). Cu-C (Fe-Mn oxide-bound) increased from 2.23 mg kg^{-1} (S1) to 8.34 mg kg^{-1} (S4), with values around 5 mg kg^{-1} at S2, S6, and S7, and ~3.5 mg kg^{-1} at S3 and S5. Cu-D (organic-bound) decreased from nearly 30 mg kg^{-1} (S1) to 9 mg kg^{-1} (S3), then gradually increased to 14.2 mg kg^{-1} (S7). Cu-E (residual) declined from 145 mg kg^{-1} (S1) to 90.6 mg kg^{-1} (S5), before increasing to 100–125 mg kg^{-1} in later stages.

Fe-A (exchangeable) decreased sharply from 0.46 mg kg^{-1} (S1) to nearly undetectable levels at S3, then increased to 1.06 mg kg^{-1} (S6) before declining again to 0.22 mg kg^{-1} (S7). Fe-B (carbonate-bound) was significantly higher at S1 and S2 (~1.2 mg kg^{-1}) than in other stages. Fe-C (Fe-Mn oxide-bound) decreased from 7.87 mg kg^{-1} (S1) to 2.43 mg kg^{-1} (S5), then increased to 5.21 mg kg^{-1} (S7). Fe-D (organic-bound) showed a similar trend, declining from 120 mg kg^{-1} (S1) to 25 mg kg^{-1} (S3) before rising to 132 mg kg^{-1} (S7). Fe-E (residual) increased from 36.5 mg kg^{-1} (S1) to 123 mg kg^{-1} (S5), then decreased to 65.8 mg kg^{-1} (S7).

Hg-A (exchangeable) remained around 6.1 μg kg^{-1} during S1–S3, increased to 6.45 μg kg^{-1} at S5, and stabilized at ~6.35 μg kg^{-1} thereafter. Hg-B (carbonate-bound) decreased from 10.4 μg kg^{-1} (S1) to 8.32 μg kg^{-1} (S3), followed by a slight but insignificant increase. Hg-C (Fe-Mn oxide-bound) was slightly above 30 μg kg^{-1} at S1, S6, and S7, but exceeded 33 μg kg^{-1} during S2–S5. Hg-D (organic-bound) increased from 24.3 μg kg^{-1} (S1) to 27.7 μg kg^{-1} (S5), then decreased to ~25 μg kg^{-1} at S7.

Mn-A (exchangeable) was highest at S6 (6.2 mg kg^{-1}), followed by S4 (4.6 mg kg^{-1}), with the lowest values at S1 and S4 (~2.6 mg kg^{-1}). Mn-B (carbonate-bound) peaked at S1 (8.02 mg kg^{-1}) and S5 (7.42 mg kg^{-1}), declining to a minimum of 1.45 mg kg^{-1} at S7. Mn-C (Fe-Mn oxide-bound) increased gradually from ~20 mg kg^{-1} (S1) to 45 mg kg^{-1} (S6). Mn-D (organic-bound) showed a more pronounced increase, rising from 10 mg kg^{-1} (S1) to 136 mg kg^{-1} (S6). Mn-E (residual) increased from 55 mg kg^{-1} (S1) to 125 mg kg^{-1} (S6), then decreased to 77 mg kg^{-1} (S7).

Pb-A (exchangeable) was relatively higher at S5 (45 μg kg^{-1}) and S6 (20 μg kg^{-1}) compared to other stages. Pb-B (carbonate-bound) gradually decreased from 2.35 μg kg^{-1} to 0.23 μg kg^{-1}. Pb-C (Fe-Mn oxide-bound) peaked at S7 (~6 μg kg^{-1}), with the lowest value at S5 (1.2 μg kg^{-1}). Pb-D (organic-bound) decreased from 52.6 μg kg^{-1} to 23.1 μg kg^{-1}, then increased again to 50.03 μg kg^{-1}. Pb-E (residual) declined slightly from S1 to S2, then gradually increased to 1.27 g kg^{-1}.

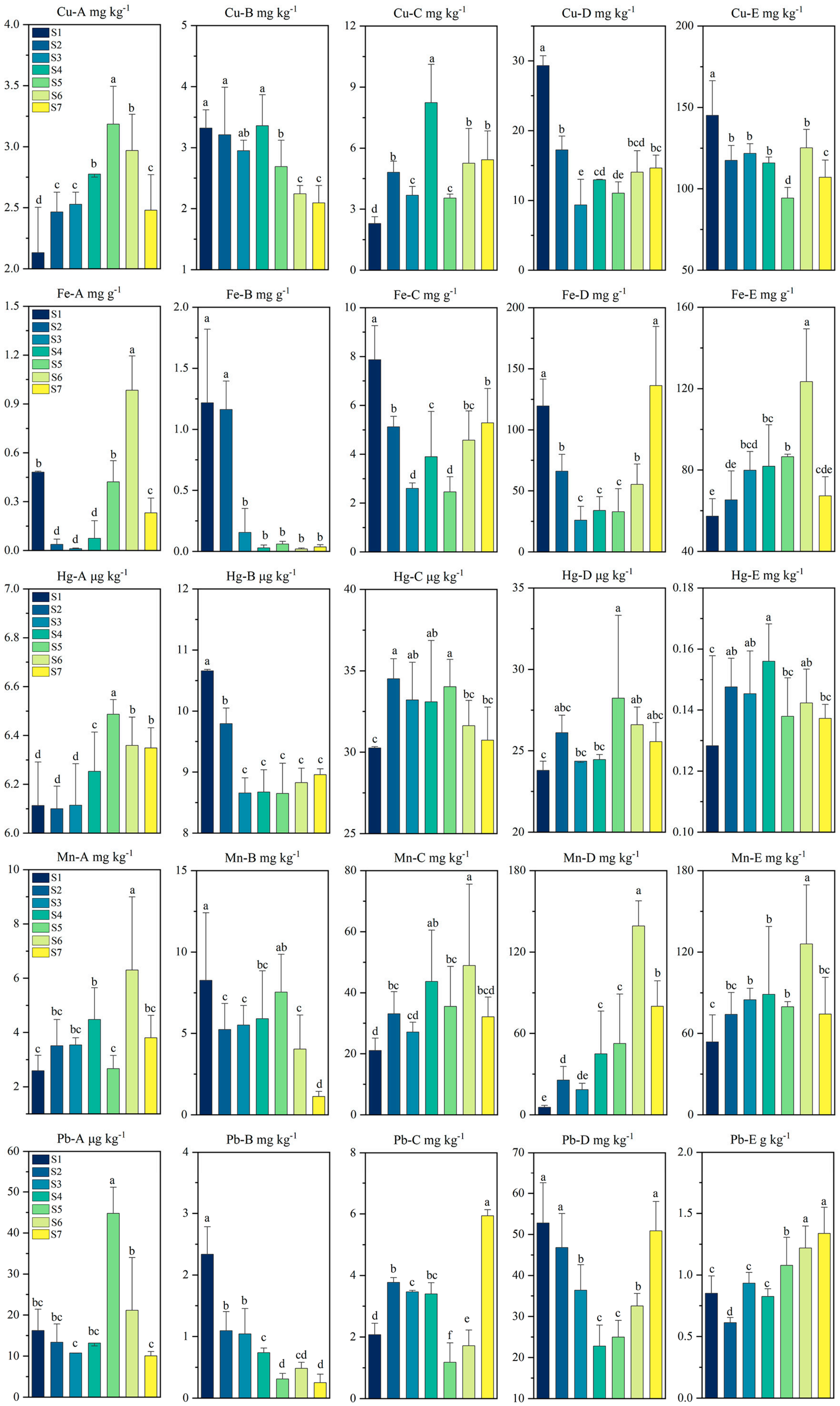
Cu-A mg kg-1
Cu-B mg kg-1
Cu-C mg kg-1
Cu-D mg kg-1
Cu-E mg kg-1
Fe-A mg g-1
Fe-B mg g-1
Fe-C mg g-1
Fe-D mg g-1
Fe-E mg g-1
Hg-A μg kg-1
Hg-B μg kg-1
Hg-C μg kg-1
Hg-D μg kg-1
Hg-E mg kg-1
Mn-A mg kg-1
Mn-B mg kg-1
Mn-C mg kg-1
Mn-D mg kg-1
Mn-E mg kg-1
Pb-A μg kg-1
Pb-B mg kg-1
Pb-C mg kg-1
Pb-D mg kg-1
Pb-E g kg-1
S1
S2
S3
S4
S5
S6
S7

Figure S4: Successional patterns of Cu, Fe, Hg, Mn and Pb fractions. A–E sequentially represent exchangeable (A), carbonate-bound (B), Fe-Mn oxide-bound (C), organic matter-bound (D), and residual (E) fractions. The different letters upon the bars in subfigures indicates significant differences between groups ($P < 0.05$, Tukey's HSD, n=10).

Supplementary 3

Based on the results described above, it is evident that heavy metals in coal gangue tailings exhibit diverse successional behaviors. Even different fractions of the same metal may display markedly distinct trends, reflecting the complex interplay of weathering, leaching, microbial mineralization, biological immobilization, plant translocation, and transformation processes. To facilitate the analysis of relationships between heavy metals and microbial community properties, dimensionality reduction was applied to the complex heavy metal fractionation data, as detailed in **S-Table 1**.

Table S1: Dimensionality reduction factors of heavy metal fractions

Fractions	PC 1	PC 2	PC 3	PC 4	PC 5	PC 6	PC 7	PC 8
AsB	0.919	0.110	0.318	-0.071	-0.004	-0.070	0.049	0.042
ZnB	0.908	0.005	0.327	-0.069	0.114	-0.009	0.081	0.063
PbB	0.899	-0.155	0.212	0.139	-0.001	-0.112	-0.025	0.030
HgB	0.885	0.057	0.126	-0.037	0.191	0.197	0.156	0.105
CuD	0.848	0.247	0.229	-0.099	0.192	-0.010	0.169	0.032
FeB	0.846	-0.218	0.023	0.019	0.154	0.316	0.204	0.012
AsA	0.837	0.119	0.405	-0.125	-0.179	-0.145	-0.007	-0.004
ZnC	0.828	0.445	-0.162	-0.128	0.039	-0.135	-0.073	0.031
CdA	0.822	0.197	-0.173	-0.054	0.218	0.067	0.240	0.150
CuA	-0.789	-0.117	0.359	-0.096	0.139	0.082	-0.080	-0.048
CdB	0.773	-0.084	0.332	0.329	-0.168	-0.007	0.057	-0.048
CdD	0.766	-0.169	0.310	0.283	-0.147	0.297	0.087	-0.052
CrB	0.762	0.291	0.437	-0.210	0.040	-0.170	0.027	0.099
CdE	0.746	-0.241	0.095	0.131	-0.271	-0.155	-0.112	-0.054
CrA	0.734	0.488	0.309	0.054	0.038	-0.203	0.010	0.091
FeC	0.732	0.423	0.053	0.042	0.380	0.074	0.014	0.086
NiB	0.728	-0.269	0.048	0.060	0.204	0.296	-0.057	-0.036
PbD	0.713	0.371	-0.303	-0.094	-0.134	0.358	-0.020	0.035
CrD	0.712	-0.488	-0.145	0.259	-0.263	0.171	-0.005	-0.010
CuE	0.672	0.178	0.251	0.486	-0.025	-0.151	-0.027	0.085
HgA	-0.665	0.281	0.280	-0.447	0.032	-0.013	0.204	-0.048
FeE	-0.641	0.185	0.442	0.460	0.036	0.116	-0.042	0.047
CrC	-0.632	0.285	0.154	-0.546	-0.074	0.009	-0.034	0.100
MnD	-0.622	0.602	0.219	0.214	0.112	0.214	0.055	-0.127
ZnA	-0.602	-0.049	0.557	-0.427	0.112	0.024	-0.045	0.111
MnE	-0.596	0.291	0.126	0.492	-0.011	0.157	-0.009	0.290

MnC	-0.592	0.146	0.142	0.332	0.445	-0.031	0.082	0.003
CrE	-0.58	0.222	0.239	0.303	-0.421	-0.227	0.098	0.404
AsC	-0.003	0.859	0.001	0.092	-0.128	-0.12	0.175	-0.276
AsD	-0.155	0.798	0.235	0.049	-0.096	0.038	0.278	-0.274
NiE	-0.036	0.763	-0.246	0.379	-0.149	0.068	-0.044	0.174
NiC	0.512	0.73	-0.381	-0.155	-0.014	0.022	-0.052	0.028
PbE	-0.446	0.723	0.091	-0.215	-0.288	-0.048	-0.083	-0.058
HgC	-0.343	-0.701	-0.146	0.006	-0.048	0.185	0.394	0.034
FeD	0.532	0.686	-0.230	-0.264	0.109	0.099	0.074	-0.006
CuB	0.473	-0.624	0.016	0.128	0.328	-0.266	-0.012	-0.029
NiA	0.276	0.584	-0.279	-0.385	-0.053	0.079	-0.158	0.093
AsE	-0.434	0.579	0.294	0.489	-0.079	0.177	0.098	-0.161
ZnE	0.442	0.565	0.317	0.189	-0.086	-0.031	-0.269	0.183
PbC	0.046	0.311	-0.926	0.001	-0.013	-0.017	-0.055	0.002
FeA	-0.133	0.537	0.733	0.087	0.035	0.196	0.077	-0.064
NiD	0.132	0.633	-0.672	-0.104	0.200	-0.059	-0.101	0.054
PbA	-0.370	-0.206	0.602	-0.510	0.023	0.150	0.096	-0.010
MnB	0.333	-0.533	0.568	-0.111	0.059	-0.203	-0.198	0.011
CdC	0.279	-0.463	-0.507	0.288	-0.245	0.466	0.036	-0.058
ZnD	0.165	-0.111	0.184	0.674	0.078	-0.013	-0.389	-0.268
MnA	-0.427	0.355	0.080	0.651	0.274	0.103	-0.032	0.063
CuC	-0.491	0.057	-0.398	0.307	0.566	-0.303	0.048	-0.037
HgD	-0.396	-0.072	0.203	-0.233	0.193	0.553	-0.325	0.357
HgE	-0.378	-0.276	-0.235	0.391	-0.099	-0.259	0.359	0.404

Note: Gray areas indicate the primary heavy metal components represented by each factor. The A–E sequentially represent exchangeable (A), carbonate-bound (B), Fe-Mn oxide-bound (C), organic matter-bound (D), and residual (E) fractions.

Among these, PC1 is primarily represented by fractions such as As-B, reflecting a general decreasing or increasing trend along the successional gradient. PC2 is mainly associated with fractions like As-C, characterized by an initial decrease followed by an increase, or vice versa, across succession stages. PC3 is represented by fractions such as Pb-C, showing significant differences in stages S5, S6, and S1 compared to other stages. Other clustered components are minor and are not discussed here.
